# Editorial: The role of STAT3 signaling pathway in tumor progression

**DOI:** 10.3389/fonc.2023.1151862

**Published:** 2023-02-17

**Authors:** Nan-Shan Chang, Kenneth KW To, Yih-Cherng Liou, Yi-Jia Li

**Affiliations:** ^1^ Graduate Institute of Biomedical Sciences, College of Medicine, China Medical University, Taichung, Taiwan; ^2^ School of Pharmacy, Faculty of Medicine, the Chinese University of Hong Kong, Hong Kong, China; ^3^ Department of Biological Sciences, Faculty of Science, National University of Singapore, Singapore, Singapore; ^4^ Department of Immuno-Oncology, Beckman Research Institute, City of Hope National Cancer Center, Duarte, CA, United States

**Keywords:** STAT3, JAK, WWOX, cancer, neurodegeneration

## Introduction

In this Introduction to our Research Topic, we would like to thank the authors for their great contributions. Also, the reviewers’ efforts are highly appreciated. Here, we updated the recent discoveries of STAT3 in supporting cancer growth and the related underlying signaling pathways, summarized what is new to the field in each article, and finally provided our perspectives in the STAT3 signaling and strategies in cancer suppression.

STAT3 is a cytosolic transcription factor, which may interact with STAT family proteins and different signaling proteins. The resulting complexes affect cellular metabolism and immune response, and promote cancer cell proliferation and differentiation (1–3). STAT3 participates in cancer promotion, chemoresistance and also enhances neurodegeneration (1–3). When STAT3 dimerizes upon stimulating with receptor associated kinases, the dimerized STAT3 becomes phosphorylated and migrates into nucleus to activate the transcription of cytokine-responsive genes (1–3). STAT3 signaling regulates glycolysis, Warburg effect, oxidative phosphorylation, ROS production, lipid and glutamine metabolism in cancer cells, immune cells and adipocytes in the tumour microenvironment, and those changes can lead to altered antitumour immune responses in tumor microenvironment (3). STAT family proteins act similarly in response to cytokines and growth factors such as IFNs, EGF, IL5, IL6, HGF, LIF and BMP2. Under physiological conditions, the expression and activation of STAT3 are finely regulated by multiple mechanisms. Aberrant regulation of the STAT3 signaling pathway has been frequently observed in cancer cells, thus leading to increased cancer migration and metastasis. STAT3 physically and/or functionally binds proteins such as STAT1, SP1, JAK1, SMAD2, EP300 and others. Database analysis shows that STAT3 activates at least 20 proteins and inactivates FOXP3, whereas it is inactivated by 13 proteins and activated by 36 proteins. The small GTPase Rac1, for example, binds and regulates the activity of STAT3 (4). PIAS3 protein specifically inhibits STAT3 (5).

## Theme articles

A complete picture of the mechanisms by which STAT3 signaling pathway is dysregulated in cancer is still missing. Lacking of tumor suppressor signaling is expected to enhance STAT3-mediated cancer growth *in vivo*. Due to its prominent role in both solid tumors and hematological malignancies, further studies are critical to better understand the role of STAT3 in tumor progression and develop strategies to inactivate STAT3. In this specific issue, we have collected five articles. We briefly described the discovery of each article and discussed as follows:

In the first article, Li et al. reported that triple negative breast cancer (TNBC) utilizes a long non-coding RNA (lncRNA) MNX1-AS1 in binding STAT3 for inducing STAT3 phosphorylation and interacting with p-JAK overexpressing and thereby promoting the growth of TNBC. Upregulation of MNX1-AS1 is shown in TNBC tumor tissues, and this correlates with poor survival outcome in TNBC patients. Notably, MNX1-AS1 upregulated the phosphorylation of STAT3 by enhancing the interaction between p-JAK and STAT3. The study suggests that MNX1-AS1 can be considered as a promising therapeutic target to TNBC. Indeed, there are many reports showing lncRNAs interact with the JAK1/STAT3 pathway that affects the progression of cancer and other types of diseases. Numerous inhibitors have been developed. Conceivably, if the presence of a specific binding motif in STAT3 for all the lncRNAs, then a small blocking molecule can be designed and used for therapeutic purposes.

In the second article, Zhao et al. reported that overexpressed tripartite motif protein 6 (TRIM6) protein enhances the migration and invasion of colorectal cancer (CRC) cells, suggesting that TRIM6 is an oncogene. Thus, knockdown of TRIM6 leads to CRC cancer suppression. TRIM6 works in signaling with STAT3 and the suppressor of cytokine signaling 2 (SOCS2). Convincing evidence shows that TRIM6 expression is positively associated with STAT3 phosphorylation and negatively correlated with SOCS2 expression. The authors suggest that TRIM6 is a potential therapeutic target for CRC metastasis. Indeed, STAT3 participates in numerous signaling pathways, and these pathways drive essentially to the ultimate destination to support cancer growth. For example, a recent report showed that BGN/FAP/STAT3 positive feedback loop mediated mutual interaction between tumor cells and mesothelial cells contributes to peritoneal metastasis of gastric cancer (6). Similarly, activating the Rac1-STAT3 pathway leads to downregulation of CYRI–B–promoted migration, invasion and EMT in gastric cancer (4).

In the third article, Pan et al. reported that ubiquitin-conjugating enzyme E2 D3 (UBE2D3) is highly expressed in glioma and the expression positively correlates with glycolysis, apoptosis, and STAT3 pathway, as determined by the Cancer Genome Atlas-Glioblastoma multiforme (TCGA-GBM) dataset. Knockdown of UBE2D3 leads to reduced glioma cell survival and increased apoptosis due to suppression of glycolysis and STAT3 phosphorylation *in vitro* and *in vivo*. Mechanistically, UBE2D3 binds SHP-2 for ubiquitination, leading to activation of the STAT3 pathway for glioma proliferation. In other word, SHP-2 counteracts the function of UBE2D3 in regulating glioma cell survival.

In the fourth article, Zhao et al. demonstrated that an FDA approved PARP1 inhibitor, Niraparib, inhibits ovarian and pancreatic ductal adenocarcinoma (PDAC) tumor cell growth, independently of BRCA mutations. Unlike the STAT3 activator Olaparib (another FDA approved PARP1 inhibitor), Niraparib inhibits STAT3 activity in ovarian and PDAC cancer cell lines and patient tumors, and thereby induces cancer cell apoptosis. The authors suggest that Niraparib inhibits pSTAT3 by interfering with SRC tyrosine kinase. Collectively, this study provides a tentative mechanism underlying Niraparib’s ability to induce tumor cell apoptosis without BRCA mutations, suggesting the potential use of Niraparib for treating PDAC patients regardless of BRCA status.

In the last article, Lu et al. reported that STAT3 participates in angiogenesis under chronic stress. Chronic stress increases the levels of hormones and induces behavioral changes in human. By using isoprenaline, the agonist of β2-adrenergic receptor (β2-AR), to stimulate chronic stress, the authors showed that isoprenaline promotes VEGF autocrine of HUVEC cells, which correlates with plexinA1 and VEGFR2 upregulation. Consequently, the event leads to activate the phosphorylation of JAK2-STAT3 pathway needed for cancer angiogenesis. Together, cancer-induced chronic stress probably activates the plexinA1/VEGFR2-JAK2-STAT3 signal transduction pathway for angiogenesis in endothelial cells.

## Conclusion

Accumulating evidence revealed that the JAK1 (or JAK2)/STAT3 pathway plays a key role in linking numerous upstream stimulators for promoting cancer growth, migration, invasion and metastasis, disordered metabolism, and neurodegeneration (1–3). STAT3 regulators include AURKA, BCL3, JAK3, KPNB1, NAMPT, NFAT5, PIM3, ROCK1, SIX1, TPX2, WWOX, and its targets are BATF3, IRF4, miR135b, miR21, RORC (7). Without doubt, the regulatory control of the signaling events are very complicated. In the collected articles, newly discovered stimulators include lncRNAs (Li et al.), SOCS2/TRIM6 (Zhao et al.), UBE2D3/SHP-2 (Pan et al.), and plexinA1/VEGFR2 (Lu et al.). A novel finding shows an FDA approved PARP1 inhibitor, Niraparib, can inhibit ovarian and PDAC progression through STAT3 inhibition (Zhao et al.). We also described the newly discovered BGN/FAP/STAT3 and the Rac1-STAT3 signaling pathways that induce cancer migration and metastasis (4, 6, 8). What’s lacking is how the downstream signaling pathways can integrate the diverse signals, so as to support cancer cell growth and push the progression of neurodegeneration and metabolic disorder.

In our perspective views, metastatic cancer cells, possessing a dysregulated JAK1/STAT3 pathway, frequently exhibit loss of tumor suppressors such as WWOX (WW domain-containing oxidoreductase). Indeed, loss of WWOX allows cancer cells to undergo metastasis (9). WWOX overexpression binds JAK2 and suppresses STAT3 phosphorylation (9). WWOX not only restricts cancer progression but also limits neurodegeneration (10–13). *WWOX* gene deficiency induces neural diseases, metabolic disorders and early death in newborn infants (11). Whether the metabolic disorders in *WWOX*-deficient newborn infants are due to dysregulated JAK1/STAT3 pathway remains to be established. Indeed, the development of metabolic disorder and neural diseases probably occurs in the embryonic stage (14). Without WWOX protein, newborn mice survive approximately 3 weeks (14). Presence of aggregated proteins, including upstream TRAPPC6AΔ, TIAF1 and SH3GLB2 as aggregation leaders and downstream amyloid beta and tau for final aggregation, are found in the brain hippocampus and cortex of two-week-old *Wwox*-deficient mice (12, 13). These mice have no apparent growth of cancer cells. Whether and how the JAK1/STAT3 pathway participates in the neurodegenerative event and early death remains to be established.

Downregulation of WWOX protein in the hippocampus and cortex in the mid-aged humans may lead to Alzheimer’s disease during old age, which is due in part to aggregation of initiator proteins such as TRAPPC6AΔ, TIAF1 and SH3GLB2 (10, 12, 13). While STAT3 is also involved in neurodegeneration (2), we assume that downregulation of WWOX leads to aggregation of JAK1 or JKA2 that allows STAT3 to exert unwanted events in neurodegeneration. Cancer cells may follow the similar pattern to undergo increased malignancy and metastasis.

## Author contributions

N-SC initiated writing the original manuscript, revised, proof read, discussed with co-authors, and finalized the manuscript. KT, Y-JL and Y-CL contributed, in part, to writing, revised and proof read the manuscript. All authors contributed to the article and approved the submitted version.
